# Glucuronolactone improves lung injury caused by PRRSV and DON co-challenge by enhancing the Nrf2-mediated antioxidant capacity in weaning piglets

**DOI:** 10.1186/s13567-025-01596-8

**Published:** 2025-08-05

**Authors:** Jing Hou, Chenbin Cui, Jing Wu, Min Tian, Qi Lu, Shilong Liu, Guohao Ye, Chaoyang Tian, Jiaxi Tang, Kaiguo Gao, Li Wang, Zongyong Jiang, Yueqin Qiu, Xuefen Yang

**Affiliations:** 1 State Key Laboratory of Swine and Poultry Breeding, Guangzhou, 510640 China; 2 Key Laboratory of Animal Nutrition and Feed Science in South China, Ministry of Agriculture and Rural Affairs, Guangzhou, 510640 China; 3 Guangdong Provincial Key Laboratory of Animal Breeding and Nutrition, Guangzhou, 510640 China; 4 Institute of Animal Science, Guangdong Academy of Agricultural Sciences, Guangzhou, 510640 China

**Keywords:** Glucuronolactone, deoxynivalenol, PRRSV, oxidative stress, lung injury, Nrf2, piglets

## Abstract

In the pig industry, both deoxynivalenol (DON) challenge and porcine reproductive and respiratory syndrome virus (PRRSV) infection can negatively impact the growth performance of pigs, thereby impairing the healthy development of the industry. Glucuronolactone (GLU) has been shown to be a potent antioxidant that helps alleviate oxidative stress. Therefore, this study aimed to investigate the effect of GLU on oxidative stress and lung injury induced by co-challenge with PRRSV and DON. Eighteen weaned piglets were randomly divided into three groups: control (CON), DON, and DON + GLU. After DON and/or GLU treatment for two weeks, all pigs were intramuscularly injected with PRRSV and treated with DON and/or GLU for another three weeks. Three weeks post-PRRSV infection, piglets in the DON group exhibited impaired growth performance, severe lung injury, and elevated viral loads. By contrast, piglets in the DON + GLU group showed improved growth performance and lung health, as well as reduced viral loads. GLU also inhibited inflammation, excessive autophagy and apoptosis induced by PRRSV and DON co-challenge in both porcine lung and MARC-145 cells, as indicated by reduced expression of pro-inflammatory factors, autophagy marker LC3, and apoptosis-related markers. Importantly, GLU can promote the phosphorylation and nuclear import of Nrf2, thereby enhancing antioxidant capacity and alleviating oxidative stress induced by PRRSV and DON co-challenge. Nrf2 inhibitor ML385 abolished the protective effect of GLU on inflammation and oxidative stress triggered by PRRSV and DON co-challenge. These findings suggest that GLU could mitigate oxidative stress to alleviate lung injury induced by PRRSV and DON co-challenge via activating the Nrf2 pathway, highlighting its potential as a dietary supplement in the pig industry.

**Supplementary Information:**

The online version contains supplementary material available at 10.1186/s13567-025-01596-8.

## Introduction

Deoxynivalenol (DON) is a trichothecene mycotoxin produced by Fusarium species, including *Fusarium graminearum* and *Fusarium culmorum*. It is commonly found in cereal grains such as wheat and maize [1].

Contamination of animal feed with DON leads to significant global economic losses, as well as severe health risks for livestock [2]. In the pig industry, chronic exposure to DON can severely impair growth performance and overall health. Symptoms of DON exposure in pigs may include reduced feed intake and efficiency, vomiting, diarrhoea, and, in severe cases, mortality [3].

Porcine reproductive and respiratory syndrome virus (PRRSV) is a highly contagious RNA virus that causes severe respiratory disease in pigs, particularly piglets [4]. The emergence of the pathogenic HP-PRRSV strain in 2006 resulted in millions of deaths within the pig industry [5]. PRRSV infection can lead to significant pathological changes in the immune organs and circulatory system of infected pigs [6]. When PRRSV enters and replicates within cells, it can cause necrosis, cellular breakdown, and the release of a large number of subviral particles. These particles then enter the lymphatic circulation and spread to immune organs such as lymph nodes and the spleen, leading to damage in these tissues and organs [7]. Research has shown that feed contaminated with DON worsens PRRSV infections, further reducing growth performance and survival rates in pigs [8]. Given the high prevalence of PRRSV and DON contamination in the swine industry, it is crucial to explore effective strategies to mitigate the combined negative effects of these two challenges for the healthy development of the pig industry.

Antibiotics have traditionally been used to manage disorders in swine production. However, their overuse has raised concerns about bacterial resistance and has led to increasing restrictions on their use in porcine feed. As a result, it is crucial to explore alternative, environmentally friendly nutritional regulation strategies to ensure the healthy development of the swine industry [9, 10].

Glucuronolactone (GLU) is a hepatoprotective agent recognised for its detoxifying and immune-modulating properties. Research has shown that GLU effectively protects against liver disorders due to its antioxidative activity [11]. Given the importance of redox homeostasis in modulating oxidative stress, dietary GLU has the potential to serve as a nutritional intervention to reduce oxidative damage caused by the co-challenge of PRRSV and DON in the pig industry.

This study aims to investigate the effects of GLU on co-challenges with PRRSV and DON using 21-day-old weaned piglets as an in vivo model, along with the African green monkey kidney cell line (MARC-145 cells) as an in vitro model. Additionally, we explore the potential mechanisms through which glucuronolactone may mitigate lung injury induced by PRRSV and DON co-challenge. These findings provide a scientific basis for considering glucuronolactone as a nutritional intervention in pig production.

## Materials and methods

### Animal experimental design, clinical monitoring, and sample collection

All animal procedures in this study received approval from the Animal Care and Use Committee of the Guangdong Academy of Agricultural Sciences (Approval No. 2024011). The experiment was carried out at the Institute of Animal Health, Guangdong Academy of Agricultural Sciences.

Weaned piglets (Duroc × Landrace × Yorkshire) with an initial body weight of 7.41 ± 0.15 kg and an age of 21 days were randomly assigned to three experimental groups (*n* = 6): the CON group (PRRSV infection without treatment for DON and GLU), the DON group (PRRSV infection with DON challenge), and the DON + GLU group (PRRSV infection with both DON and GLU treatment). An equal number of males and females were included in each group.

To determine the optimal dosage of GLU in vivo, we based our approach on a previous study that showed 200 mg/kg of GLU improved the growth performance of weaned piglets [12]. We hypothesised that this dosage would help mitigate the adverse effects of DON in PRRSV-infected piglets. Thus, for this experiment, we selected a moderate dose of 200 mg/kg GLU for a more detailed mechanistic investigation.

The basal diet, which served as the control diet, was formulated according to the 2012 nutrient recommendations from the National Research Council (NRC) (Additional file 1). The piglets in the DON group received a basal diet supplemented with 2.0 mg/kg of DON. The DON + GLU group received a basal diet supplemented that included both 2.0 mg/kg of DON and 200 mg/kg of GLU. All piglets were fed four times daily (08:00, 11:00, 15:00, and 18:30) for a duration of two weeks. They were housed in standard high-raised nursery pens with ad libitum access to feed and water.

Following the feeding regimen, each piglet in the CON, DON, and DON + GLU groups received an intranasal inoculation of 2 mL PRRSV solution (10^5^ TCID_50_/mL).

Three weeks after the PRRSV infection, a necropsy and gross pathological examination of the lungs was conducted immediately following euthanasia using sodium pentobarbital (40 mg/kg body weight). Throughout the experiment, clinical symptoms were closely monitored and evaluated using a detailed scoring system [13].

Average daily gain (ADG) was determined by weighing the piglets at the beginning and end of the experiment following a 12-h overnight fast. After the experiment, serum and lung samples were collected and stored in liquid nitrogen at − 80 ℃ for further analysis.

### Serum immunoglobulins and cytokine determination

The serum from the piglets was separated, and levels of cytokines (IL-1β, IL-10, IFN-α, IFN-β, and IFN-γ) and immunoglobulin (IgA, IgG, and IgM) were measured using commercial ELISA kits (MEIMIAN, Jiangsu, China), according to the manufacturer’s instructions.

### Serum biochemical parameters measurement

Serum biochemical parameters, including glucose (GLU), total protein (TP), albumin (ALB), urea (URE), alanine aminotransferase (ALT), aspartate aminotransferase (AST), and creatinine (CRE), were analysed using commercial assay kits (Biosino Biotechnology and Science Inc., Beijing, China). The analyses were performed on an automated biochemical analyser (Selectra Pro XL, EliTechGroup, Puteaux, France).

### Organ index

After the piglets were exsanguinated, the weights of the heart, liver, spleen, lungs, and kidneys were recorded to calculate the organ index. The organ index (%) was determined using the following formula:

$${\text{Organ}}\,{\text{index }}\left( \% \right)\, = \,\left( {{\text{organ}}\,{\text{weight}}/{\text{pre}} - {\text{slaughter}}\,{\text{live}}\,{\text{weight}}} \right)\, \times \,{1}00$$

### Haematoxylin and eosin, immunohistochemistry, and immunofluorescence assay

Lung tissues were collected and fixed in 4% paraformaldehyde (Biosharp Life Sciences, Hefei, China) at room temperature for 48 h. After this, routine paraffin embedding was performed. The resulting tissue blocks were sectioned into slices that were 8–10 μm-thick. Following dewaxing, histopathological evaluation was conducted using haematoxylin and eosin (H&E) staining. A veterinary pathologist, who was blinded to the experimental groups (CON, DON, and DON + GLU), evaluated the specimens according to a standardised scoring system [13].

Immunohistochemistry was performed using a caspase-3 monoclonal antibody (1:1000 dilution) (Cell Signalling Technology, MA, USA) to detect apoptotic cells. Furthermore, PRRSV N protein expression in lung tissue was assessed through an immunofluorescence assay using a PRRSV N monoclonal antibody (1:500 dilution) (Bioss, Beijing, China) and a corresponding fluorescently labelled secondary antibody.

### Viruses, cell lines, and compounds

The PRRSV genotype 2 (GD-ZJ, GenBank: MF772778.1, American strain) and the MARC-145 cell line were generously provided by Professor Song’s laboratory [14]. The MARC-145 cell line, known for its susceptibility to PRRSV, was cultured in Dulbecco’s Modified Eagle Medium (DMEM; Gibco, UT, USA) that was supplemented with 10% foetal bovine serum (FBS; Biological Industries, Kibbutz Beit Haemek, Israel), in addition to 100 IU/mL penicillin and 100 µg/mL streptomycin. The cells were maintained at 37 °C in a 5% CO_2_ incubator. Deoxynivalenol (CAS: 51481-10-8, 98% purity) and glucuronolactone (CAS: 32449-92-6, 99% purity) were obtained from Sigma-Aldrich (St. Louis, MO, USA). ML385 was purchased from Glpbio (Montclair, CA, USA).

### Viral titre determination

Adherent MARC-145 cells were trypsinised, centrifuged, and resuspended in PBS (Biosharp Life Sciences, Hefei, China). The cells were then centrifuged again at 1000 rpm for 3 min. The cell concentration was adjusted to 1 × 10^5^ cells/mL using DMEM medium. A volume of 100 μL of this cell suspension was added to each well of a 96-well cell culture plate (Biosharp Life Sciences, Hefei, China) and incubated at 37 °C in a 5% CO₂ atmosphere for 48 h.

After the incubation period, the medium was discarded, and the cells were washed three times with PBS. Subsequently, 100 μL PRRSV solution was added to each well at titres of 10^–1^ – 10^–10^ TCID_50_/mL. Normal MARC-145 cells were included as a control to determine the optimal viral titre required to induce cytopathic effects.

### Determination of GLU toxicity in MARC-145 cells

MARC-145 cells in 96-well plates were washed three times with PBS. Following this, 100 μL of GLU solution was added to each well at concentrations of 10, 50, 100, 500, and 1000 μg/mL, with each concentration represented in triplicates. The cells were then incubated for 48 h at 37 °C in a 5% CO₂ incubator.

After the incubation period, 10 μL CCK-8 solution was added to each well, and the cells were incubated for an additional 4 h. Subsequently, 100 μL DMSO (Aomabio, Guangzhou, China) was added to each well, and the absorbance was measured at 450 nm using a microplate reader. Cells that did not receive GLU treatment served as the blank control. The 50% cytotoxic concentration (CC_50_) was calculated using GraphPad Prism 7.0 software, with medium alone serving as the negative control.

### Determination of DON toxicity in MARC-145 cells

MARC-145 cells were cultured in 96-well plates and washed three times with PBS. Following the washes, 100 μL DON solution at concentrations of 50, 100, 250, 500, and 1000 ng/mL was added to each well, with triplicates for each concentration. The cells were then incubated for 48 h at 37 °C in a 5% CO₂ incubator.

After the incubation period, 10 μL CCK-8 solution was added to each well, and the cells were incubated for an additional 4 h. Subsequently, 100 μL DMSO was added to each well, and the absorbance was measured at 450 nm using a microplate reader. Cells that did not receive DON treatment were used as a blank control.

### Impact of DON on the viability of MARC-145 cells infected with PRRSV

MARC-145 cells were seeded into 96-well plates to create a monolayer. After removing the medium, the cells were washed with PBS and infected with 100 μL of virus solution at the optimal viral titre (as described in the section “Viral titre determination”) for 2 h at 37 °C in a 5% CO₂ incubator. Following the incubation, the cells were washed again with PBS and treated with 100 μL of DON solution at various concentrations. The cells were then incubated for an additional 48 h at 37 °C in a 5% CO₂ atmosphere. To assess cell viability, CCK-8 solution was added to each well, and absorbance was measured at 450 nm using a microplate reader. The PRRSV-infected group that did not receive DON treatment served as the control.

### Assessing the effect of GLU on the viability of MARC-145 cells co-challenged with PRRSV and DON

MARC-145 cells were seeded into 96-well plates and incubated to form a monolayer. After discarding the medium, the cells were washed with PBS. Based on the GLU concentration determined in the section titled “Determination of GLU toxicity in MARC-145 cells”, 100 μL of GLU-containing medium was added to each well and incubated for 4 h at 37 °C in a 5% CO₂ incubator. The cells were then washed twice with PBS.

Next, 100 μL of virus solution (at the optimal viral titre described in the section “Viral titre determination”) was added to each well, and the cells were incubated for 2 h at 37 °C in a 5% CO₂ atmosphere. After another wash with PBS, 100 μL DON solution was added to each well. Following a 48-h incubation at 37 °C with 5% CO₂, CCK-8 solution was added, and cell viability was assessed by measuring the OD at 450 nm using a microplate reader. The untreated group served as the control to evaluate the effect of GLU on the viability of cells co-challenged with PRRSV and DON.

### RNA extraction and quantitative real-time PCR

Lung tissue or cell samples were processed to extract total RNA using TRIzol reagent (Invitrogen, Carlsbad, CA, USA). The concentration and purity of the RNA were assessed using a NanoDrop ND-1000 Spectrophotometer (Nano-Drop Technologies, Rockland, DE), and RNA integrity was evaluated through electrophoresis on 1% agarose gels.

First-strand cDNA synthesis was performed by reverse transcription of 1 μg total RNA using the PrimeScript RT reagent kit with gDNA Eraser (Takara, Tokyo, Japan). Real-time qPCR was conducted using the Bio-Rad CFX System in a final reaction volume of 20 µL. This volume comprised of 2 µL of cDNA (diluted 1:9, w/v), 10 µL of iTaq Universal SYBR Green PCR Supermix (2 ×, Bio-Rad, Hercules, CA, USA), 6.4 µL of RNase-free water, and 0.8 µL of each forward and reverse primer (10 µM/L) as detailed in Additional file 2.

The qRT-PCR program included an initial denaturation step at 95 °C for 3 min, followed by 40 cycles of denaturation at 95 °C for 15 s, annealing at 58 °C for 15 s, and extension at 72 °C for 35 s. Amplification products were analysed using the CFX Connect Real-Time System (Bio-Rad, CA, USA).

Fold changes in the target genes were normalised against β-actin using the 2^−ΔΔCt^ method [15]. The copy number of the PRRSV N was determined using real-time PCR with an absolute quantitation method [16]. Sample processing and data acquisition were performed by technicians who were unaware of the experimental groups.

### Western blot assay

Lung tissue or cell samples were collected and homogenised in cooled lysis buffer at a 1:10 (w/v) ratio. The samples were then centrifuged at 12 000 × *g* for 5 min at 4 °C to obtain the supernatant. Protein concentrations were measured using the BCA protein assay kit (Beyotime Biotechnology, Shanghai, China). Approximately 40 μg of protein was separated by electrophoresis using either a 10% or 12% SDS-PAGE gel (Beyotime, Shanghai, China) and transferred onto a polyvinylidene fluoride (PVDF) membrane (Millipore, Bedford, MA, USA) at 200 mA for 2 h.

After transfer, the membranes were blocked with 5% skim milk powder for 2 h at room temperature. They were then incubated overnight at 4 °C with primary antibodies. The following primary antibodies were used: anti-PRRSV N-protein (1:500, Bioss, Beijing, China), anti-β-actin (1:1000, Santa Cruz, CA, USA), anti-IL-1β (1:1000, Proteintech, Wuhan, China), anti-IL-6 (1:1000, Proteintech, Wuhan, China), anti-P62 (1:1000, Abmart, Shanghai, China), anti-FUNDC1 (1:1000, HUABIO, Hangzhou, China), anti-LC3A/B (1:1000, Abmart, Shanghai, China), anti-Bax (1:1000, Abmart, Shanghai, China), anti-Bcl-2 (1:1000, Abmart, Shanghai, China), anti-caspase-3 (1:2000, Cell Signaling Technology, MA, USA), anti-Nrf2 (1:000, Abmart, Shanghai, China), anti-phospho-Nrf2 (1:1000, Abmart, Shanghai, China), anti-Keap1 (1:2000, Proteintech, Wuhan, China), anti-HO-1 (1:1000; Cell Signaling Technology, MA, USA), anti-histone H3 (1:2000, Abmart, Shanghai, China) and anti-GAPDH (1:1000, Proteintech, Wuhan, China).

Protein bands were visualised using an enhanced chemiluminescence (ECL) detection system (ChemiDoc XRS, Thermo Fisher Scientific, Wilmington, DE, USA). Densitometric analysis of band intensity was performed using ImageJ software (ImagePro Plus 6.0, Media Cybernetics, Inc., Rockville, MD, USA), and relative protein expression was normalised to β-actin or GAPDH as loading controls.

### Antioxidant capacity evaluation

Malondialdehyde (MDA), total antioxidant capacity (T-AOC), superoxide dismutase (SOD), catalase (CAT), and glutathione peroxidase (GSH-PX) activities were measured using commercial kits from Nanjing Jiancheng Bioengineering Institute (Nanjing, China). Lung tissue samples were homogenised in physiological saline, and the supernatants were collected after centrifugation for further analysis. The final data were normalised to the total protein concentration in the tissues.

### Reactive oxygen species (ROS) detection

Cells were seeded into 6-well plates according to the specified treatment conditions. After the treatment, the cells were collected using trypsin digestion, washed with PBS, and transferred to 1.5 mL centrifuge tubes. They were centrifuged at 1000 rpm for 5 min. Some of the control cells were resuspended in complete medium without any staining. For the negative control, other cells were discarded from the supernatant and resuspended in 200 μL serum-free basal medium containing 10 μmol/L DCFH2-DA (Sigma-Aldrich, MO, USA). The cells were mixed and incubated at room temperature, protected from light, for 30 min. following the incubation, the cells were centrifuged at 1000 rpm for 5 min, and the supernatant was discarded. The cells were washed twice with basal medium. Finally, the cells were resuspended in 200 μL of serum-free basal medium, and the fluorescence intensity at 488 nm was measured using flow cytometry.

### Statistical analysis

Statistical analyses were performed using GraphPad Prism 8.0. Results are presented as the mean ± SEM for both in vitro and in vivo animal experiments. The normality of data distribution was evaluated using the Shapiro–Wilk test. Differences between groups were analysed using one-way analysis of variance (ANOVA) followed by Tukey’s multiple comparison test. A statistical significance level of *P* < 0.05 was established.

## Results

### GLU improves clinical symptoms and enhances growth performance in PRRSV and DON co-challenged weaned piglets

**Figure 1:**
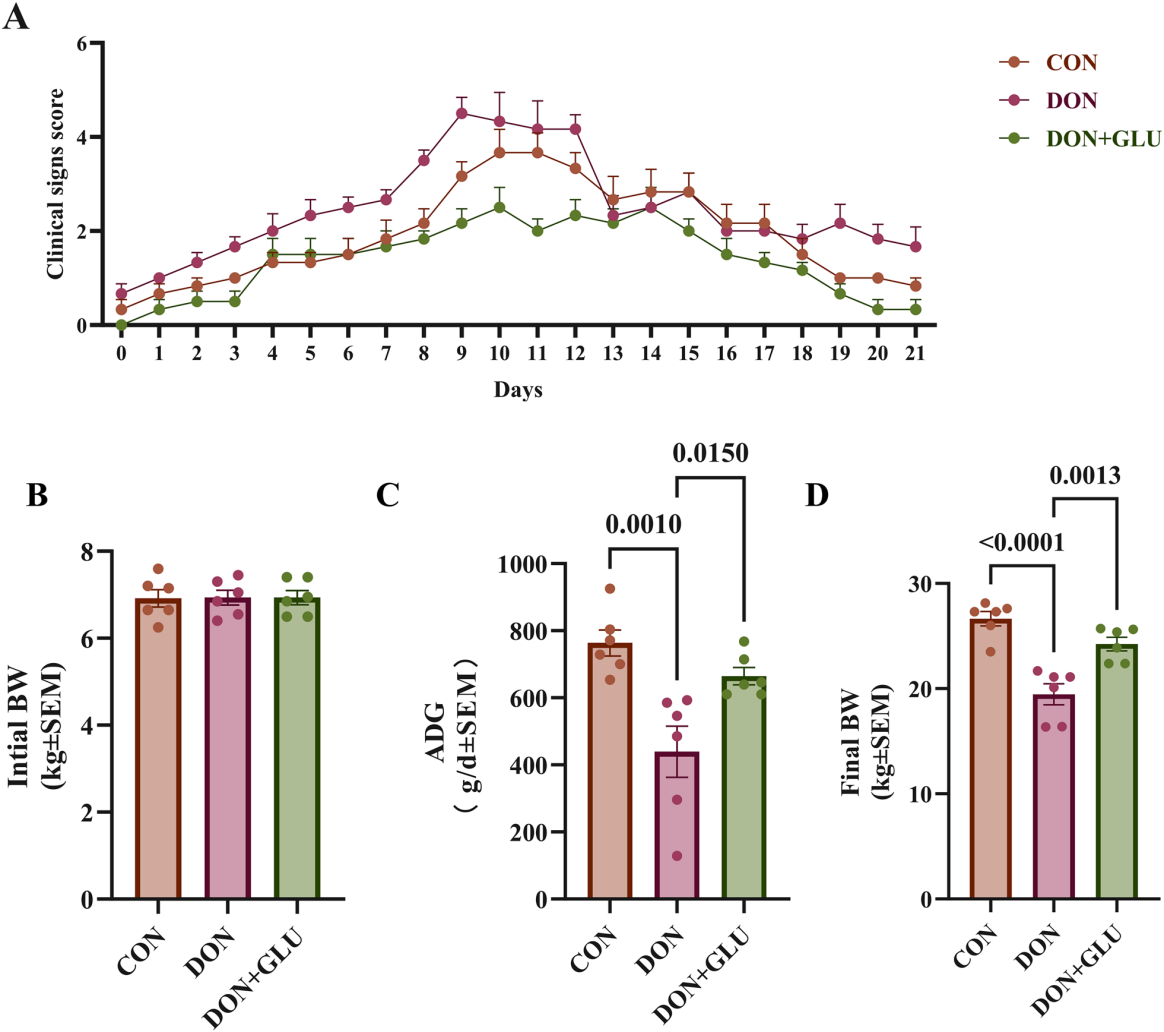
**GLU ameliorates clinical symptoms and enhances growth performance in PRRSV and DON co-challenged weaned piglets**. **A** Clinical score. **B**–**D** Growth performance of piglets in different groups. The data are expressed as mean ± SEM (*n* = 6). CON, control; DON, deoxynivalenol; DON + GLU, combination of deoxynivalenol and glucuronolactone; BW, body weight; ADG, average daily gain.

After PRRSV infection, the piglets exhibited typical clinical signs, including tachypnoea, coughing, lethargy, decreased appetite, delayed responses, and swollen eyelids (Fig. 1A). Compared to the control group, piglets in the DON group displayed significantly more severe clinical symptoms, which included increased nasal and ocular secretions, conjunctival oedema, and overall clinical deterioration. In contrast, piglets in the DON + GLU group showed considerably milder symptoms, evidenced by a notable reduction in clinical scores and slower disease progression. Piglets receiving additional GLU had lower rates of conjunctival oedema and had less nasal and ocular discharge compared to those in the DON group.

Figures 1B, C and D illustrate the growth performance of piglets across different groups. It has been established that PRRSV infection can cause a reduction in the ADG of piglets [17–20].

When compared to the control group, exposure to DON significantly decreased the ADG (*P* = 0.0010) and final body weight (*P* < 0.0150) of the piglets. However, with DON exposure, the addition of GLU significantly improved the ADG (*P* = 0.0150) and final body weight (*P* = 0.0013) compared to the group solely exposed to DON.

These findings suggest that GLU supplementation reduces both the clinical symptoms and growth suppression caused by PRRSV and DON co-challenge in piglets.

### GLU attenuates pulmonary lesions in PRRSV- and DON-co-challenged piglets

**Figure 2:**
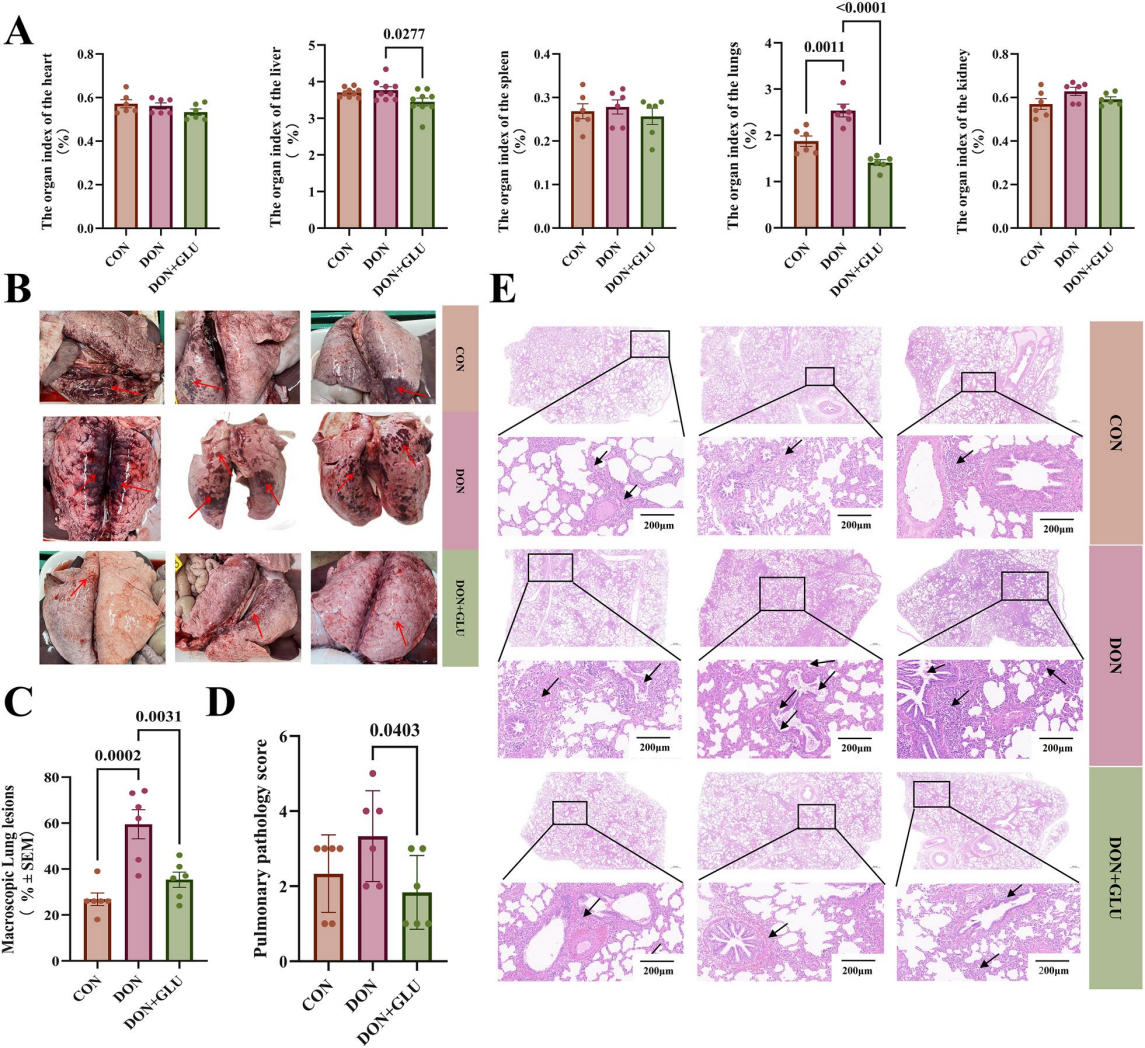
**GLU attenuates pulmonary lesions in PRRSV and DON co-challenged piglets**. **A**, **B** Macroscopic lung lesion changes in PRRSV- DON co-challenged piglets with GLU treatment. **C**–**E** The lungs of piglets were collected, and sections were prepared to stain with haematoxylin and eosin (H&E). Black arrows indicate the histopathological alterations, including progressive loss of lung architecture, thickening of the interlobular septa, and infiltration of lymphocytes (scale bar = 200 μm). The data are expressed as mean ± SEM (*n* = 6). CON, control; DON, deoxynivalenol; DON + GLU, combination of deoxynivalenol and glucuronolactone.

We then evaluated the changes in organ index. As shown in Fig. 2A, the lung index was significantly higher in the DON group compared to the CON group (*P* = 0.0011). In contrast, piglets in the DON + GLU group displayed a significantly lower lung index (*P* < 0.0001) and liver index (*P* = 0.0277) compared to the DON group.

Macroscopic examination of lung tissues revealed lesions in piglets infected with PRRSV. These lesions were characterised by dark discolouration, extensive parenchymal consolidation, and congestion. Piglets in the DON group exhibited more severe lung lesions compared to those in the CON group (*P* = 0.0002) (Figs. 2B and C). However, piglets in the DON + GLU group showed a significant reduction in lung lesions, demonstrating smaller areas of consolidation and fewer haemorrhagic points (*P* = 0.0031) (Figs. 2B and C).

Additionally, histopathological analysis confirmed the increased lung injury in piglets from the DON group, which was indicated by moderate thickening of the alveolar walls, enlarged interalveolar septa, and intense infiltration of inflammatory cells (Figs. 2D and E). In contrast, piglets in the DON + GLU group exhibited fewer histopathological alterations, reflected by a decreased pathology score (*P* = 0.0403) (Figs. 2D and E).

We also evaluated the biochemical indicators of liver and kidney function in our study. As presented in Additional File 4, the group exposed to DON showed significantly elevated serum levels of aspartate aminotransferase (AST) compared to the control group (*P* = 0.0001). However, in the group that received DON + GLU, serum albumin (ALB) levels were significantly higher (*P* = 0.0163) compared to the DON group. Additionally, the serum concentrations of urea (URE) (*P* = 0.0135), AST (*P* < 0.0001), and alanine aminotransferase (ALT) (*P* = 0.0006) were significantly reduced in the DON + GLU group compared to the DON group.

These findings indicate that dietary GLU significantly alleviates pulmonary injury in weaned piglets co-challenged with PRRSV and DON.

### Dietary GLU suppresses viral replication and reduces the expression of the PRRSV N protein in piglets co-challenged with PRRSV and DON

**Figure 3:**
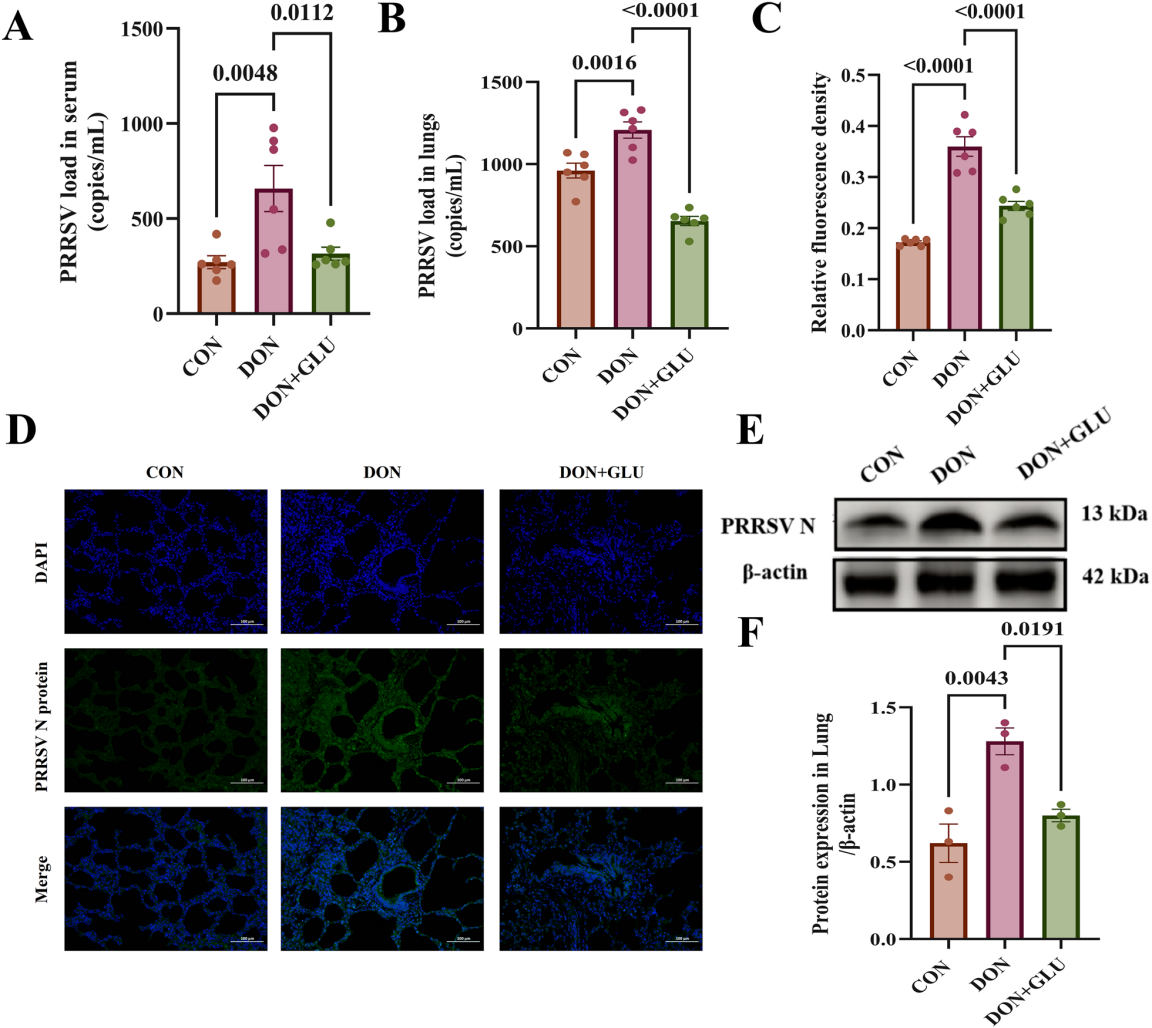
**Dietary GLU suppresses viral replication and attenuates PRRSV N protein expression in PRRSV and DON co-challenged piglets**. **A** Viral loads of PRRSV in serum calculated by absolute quantitation. **B** Viral loads of PRRSV in lungs calculated by absolute quantitation. **C**, **D** IFA analysis of protein N (green) using a rabbit PRRSV N protein polyclonal antibody, scale bar = 100 μm. **E**, **F** Expression of the PRRSV N protein in lung tissues was detected using western blotting. The data are expressed as mean ± SEM (*n* = 3 or 6). CON, control; DON, deoxynivalenol; DON + GLU, combination of deoxynivalenol and glucuronolactone.

As illustrated in Figs. 3A and B, the viral loads in porcine serum (*P* = 0.0048) and lung tissue (*P* = 0.0016) were significantly higher in the DON group compared to the CON group. In contrast, piglets in the DON + GLU group exhibited a significant reduction in viral load in both serum (*P* = 0.0112) and lung tissue (*P* < 0.0001) compared to the DON group (Figs. 3A and B).

Immunofluorescence staining of lung tissues revealed that lung tissues from piglets in the DON group displayed intense fluorescence for the PRRSV N protein (green), indicating a higher viral load than that in the CON group (*P* < 0.0001) (Figs. 3C and D). In contrast, the DON + GLU group showed a significantly weaker fluorescence signal, suggesting a marked decrease in lung viral load compared to the DON group (*P* < 0.0001) (Figs. 3C and D).

Western blot analysis was also performed to quantify the expression levels of the PRRSV N protein in the lung. Consistent with our previous findings, piglets in the DON group had significantly higher PRRSV N protein expression in the lungs compared to the CON group (*P* = 0.0043) (Figs. 3E and F). Conversely, piglets in the DON + GLU group exhibited significantly lower expression of the PRRSV N protein compared to the DON group (*P* = 0.0191) (Figs. 3E and F).

These findings indicate that dietary GLU significantly reduces the PRRSV viral load in the serum and lung tissue of piglets co-challenged with PRRSV and DON.

### Protective effects of GLU against inflammation induced by PRRSV and DON co-challenge, both in vivo and in vitro

**Figure 4:**
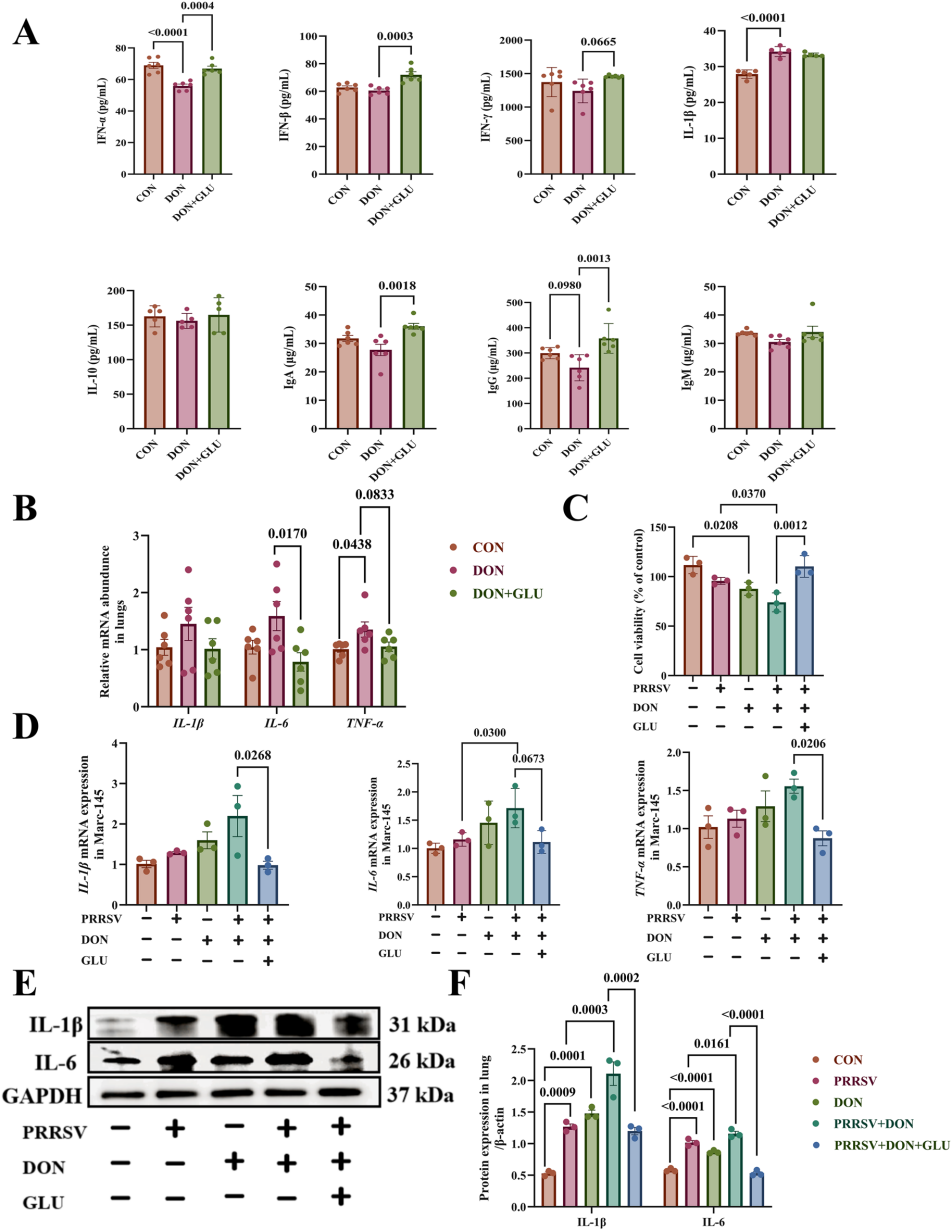
**Protective effect of GLU on inflammation induced by PRRSV and DON co-challenge in vivo and in vitro**. **A** Inflammatory cytokine and immunoglobulin levels in serum from different treatment groups. **B** Relative expression of inflammatory cytokines in the lung. **C** Effect of glucuronolactone on cell viability by PRRSV and DON co-challenge. **D** Relative expression of inflammatory cytokines in the MARC-145 cells. **E**, **F** Cytokine protein expression examined by western blot. The data are expressed as mean ± SEM (*n* = 3 or 6). *CON* control; *DON* deoxynivalenol; *DON + GLU* combination of deoxynivalenol and glucuronolactone, *IL-1β* Interleukin-1 beta, *IL-6* Interleukin-6, *IL-10* Interleukin-10, *IFN-α* Interferon-alpha, *IFN-β* Interferon-beta, *IFN-γ* Interferon-gamma, *IgA* immunoglobulin A, *IgG* immunoglobulin G, *IgM* immunoglobulin M, *TNF-α* Tumour Necrosis Factor-alpha.

Exposure to DON resulted in significantly decreased serum levels of IFN-α and significantly increased levels of IL-1β (*P* < 0.0001) compared to the CON group (Fig. 4A). In contrast, GLU supplementation significantly upregulated several key immune markers, including IFN-α (*P* = 0.0004), IFN-β (*P* = 0.0003), IgA (*P* = 0.0018) and IgG (*P* = 0.0013), compared to the DON group (Fig. 4A). To further evaluate pulmonary immune responses, we examined the mRNA expression levels of various cytokines and antiviral factors (Fig. 4B). The DON group showed significantly increased *TNF-α* mRNA expression compared to the CON group (*P* = 0.0438). Additional GLU treatment effectively suppressed *IL-6* (*P* = 0.0170). These results suggest that GLU supplementation may help mitigate the inflammatory responses induced by PRRSV and DON co-challenge in porcine serum and lung tissue.

We subsequently investigated the effect of GLU on PRRSV and DON co-challenge in vitro. To determine the optimal experimental conditions, we assessed the cytotoxicity of DON in MARC-145 cells (Additional file 5A). Our findings showed that exposure to DON at concentrations of ≥ 500 ng/mL for 48 h significantly reduced cell viability (*P* = 0.0030). Consequently, we selected 500 ng/mL of DON for subsequent experiments.

Similarly, the CCK-8 assay for GLU cytotoxicity demonstrated that GLU at a concentration of 500 µg/mL significantly enhanced cell viability (*P* = 0.0120, Additional file 4B). The calculated 50% cytotoxic concentration (CC_50_) was found to be 905.4 μg/mL (Additional file 4C). Therefore, we chose to use 500 µg/mL GLU for further analysis in the subsequent experiments.

As illustrated in Fig. 4C, exposure to DON (*P* = 0.0208) significantly reduced the viability of MARC-145 cells compared to the CON group. Additionally, co-exposure to PRRSV and DON exacerbated cell death, as indicated by the further reduction in cell viability (*P* = 0.0370). Importantly, GLU supplementation significantly restored cell viability when compared to the PRRSV + DON group (*P* = 0.0012).

To evaluate the anti-inflammatory effects of GLU, we analysed the expression of pro-inflammatory cytokines (Fig. 4D). The PRRSV + DON group showed significantly elevated levels of *IL-6* mRNA compared to the PRRSV group (*P* = 0.0300). As anticipated, GLU supplementation significantly suppressed the mRNA expression of *IL-1β* (*P* = 0.0268) and *TNF-α* (*P* = 0.0206) compared to the PRRSV + DON group.

Western blot analysis demonstrated that PRRSV infection significantly increased the protein expression levels of IL-1β (*P* = 0.0009) and IL-6 (*P* < 0.0001) compared to the CON group. Furthermore, exposure to DON also markedly elevated the expression of these proinflammatory cytokines (*P* = 0.0001, *P* < 0.0001, Figs. 4E and F). Co-challenging with PRRSV and DON further increased IL-1β (*P* = 0.0003) and IL-6 (*P* = 0.0161) protein levels compared to the PRRSV group alone. Importantly, GLU supplementation dramatically reduced the protein levels of IL-1β (*P* = 0.0002) and IL-6 (*P* < 0.0001) when compared to the PRRSV + DON group (Figs. 4E and F).

In conclusion, these results suggest that GLU supplementation effectively alleviates the severe inflammation induced by the co-challenge of PRRSV and DON in both in vivo and in vitro settings.

### GLU mitigates excessive autophagy and apoptosis induced by PRRSV and DON co-challenge

**Figure 5:**
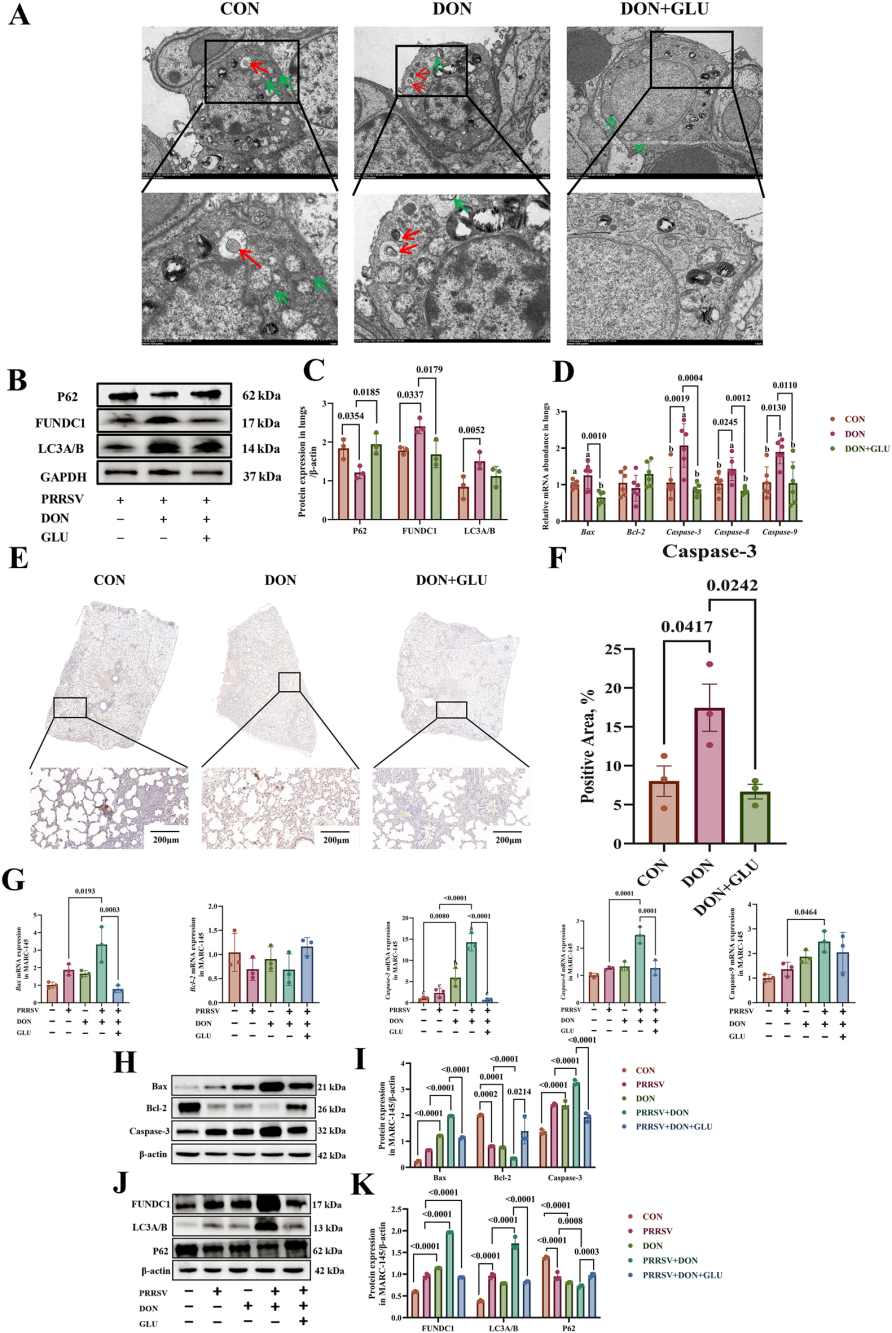
**GLU mitigates apoptosis and excessive autophagy induced by PRRSV and DON co-challenged**.** A** Transmission electron microscopy (TEM) analysis of lung ultrastructure in piglets. **B**, **C** Western blot analysis of autophagy-related protein expression in lung tissues. **D** mRNA expression of apoptosis-related genes by qPCR in lung tissues. **E–F** Expression of caspase-3 protein in lung tissues was detected by IHC with a rabbit caspase-3 protein polyclonal antibody, scale bar = 200 μm. **G** mRNA expression of apoptosis-related genes by qPCR in MARC-145 cells. **H-I** Western blot analysis of apoptosis-related protein expression in MARC-145 cells. **J-K** Western blot analysis of autophagy-related protein expression in MARC-145 cells. The data are expressed as mean ± SEM (*n* = 3). CON, control; DON, deoxynivalenol; DON + GLU, combination of deoxynivalenol and glucuronolactone. P62, sequestosome 1; *FUNDC1* FUN14 domain containing 1, *LC3A/B* microtubule-associated protein 1 light chain 3 alpha/microtubule-associated protein 1 light chain 3 beta; GAPDH, glyceraldehyde-3-phosphate dehydrogenase, *Bax* Bcl-2-associated X protein, *Bcl-2* B-cell lymphoma 2.

Transmission electron microscopy revealed significant ultrastructural damage in alveolar epithelial cells following concurrent infection with PRRSV and exposure to DON. In the CON group, type II alveolar epithelial cells displayed moderate organelle swelling, minimal autolysosome formation (green arrows), and signs of mitochondrial microautophagy (indicated by red arrows) (Fig. 5A). In contrast, the DON group showed severe cellular damage characterised by pronounced cell shrinkage and a significantly increased number of autolysosomes, suggesting excessive autophagy. Notably, in the DON + GLU group, the alveolar epithelial cells exhibited only mild organelle expansion, with fewer autolysosomes and no typical structures associated with mitochondrial autophagy (Fig. 5A).

To quantify the expression of key autophagy markers, western blot analysis was conducted. Compared to the CON group, the DON group demonstrated significantly elevated levels of FUNDC1 (*P* = 0.0337) and LC3A/B (*P* = 0.0052), along with a notable decrease in P62 expression (*P* = 0.0354), indicating heightened autophagic activity (Figs. 5B and C). In the DON + GLU group, FUNDC1 expression levels were significantly reduced (*P* = 0.0179), while P62 levels increased (*P* = 0.0185) (Figs. 5B and C). These findings suggest that GLU could help mitigate excessive autophagy induced by PRRSV and DON.

Excessive autophagy can trigger apoptosis [21]; therefore, we assessed apoptosis in the lungs of piglets next. As shown in Fig. 5D, the mRNA expression levels of *caspase-3* (*P* = 0.0019), *caspase-8* (*P* = 0.0245), and *caspase-9* (*P* = 0.0130) were significantly higher in the DON group compared to the CON group. In the DON + GLU group, the expression levels of *Bax* (*P* = 0.0010), *caspase-3* (*P* = 0.0004), *caspase-8* (*P* = 0.0012), and *caspase-9* (*P* = 0.0110) were significantly reduced compared to the DON group.

Furthermore, immunohistochemical staining was performed to confirm these findings. Lung sections from the DON group exhibited intense caspase-3-positive signals, primarily located in macrophages, alveolar epithelial cells, alveolar interstitial tissue, bronchioles, and alveolar septa (Figs. 5E and F). In contrast, caspase-3 expression was markedly reduced in the DON + GLU group.

Subsequently, we conducted MARC-145 cell experiments to further validate the protective effect of GLU on apoptosis and excessive autophagy. Compared to the CON group, the DON group showed a significant increase in *caspase-3* mRNA expression (*P* = 0.0080), indicating enhanced apoptosis (Fig. 5G). Additionally, the PRRSV + DON group had significantly higher expression of *Bax* (*P* = 0.0193), *caspase-3* (*P* < 0.0001), *caspase-8* (*P* = 0.0001), and *caspase-9* (*P* = 0.0464) compared to the PRRSV group, confirming that DON exacerbates apoptosis.

Notably, in the PRRSV + DON + GLU group, the expression levels of *Bax* (*P* = 0.0003), *caspase-3* (*P* < 0.0001), and *caspase-8* (*P* = 0.0001) were significantly reduced compared to the PRRSV + DON group (Fig. 5G). Western blot analysis supported these trends at the protein level. PRRSV infection significantly downregulated Bcl-2 protein expression (*P* = 0.0002) compared to the CON group. Furthermore, exposure to DON markedly upregulated the expression of Bax and caspase-3 proteins (*P* < 0.0001) while simultaneously suppressing Bcl-2 levels (*P* = 0.0001) (Figs. 5H and I).

Moreover, in comparison to the PRRSV group, the PRRSV + DON group demonstrated a further increase in the expression of Bax and caspase-3, along with a significant decrease in Bcl-2 expression (*P* < 0.0001). However, GLU supplementation notably reduced the levels of Bax and caspase-3 (*P* < 0.0001) and restored Bcl-2 expression in the PRRSV + DON + GLU group when compared to the PRRSV + DON group (*P* = 0.0214) (Figs. 5H and I).

Similarly, Western blot analysis showed that PRRSV infection significantly increased the protein expression levels of LC3A/B, while reducing P62 expression compared to the CON group (*P* < 0.0001). In contrast, exposure to DON significantly elevated FUNDC1 protein expression and simultaneously decreased P62 levels (*P* < 0.0001). Additionally, compared to the PRRSV group, the PRRSV + DON group exhibited further elevation in FUNDC1 and LC3A/B levels (*P* < 0.0001), along with a significant reduction in P62 expression (*P* = 0.0008). Interestingly, in the PRRSV + DON + GLU group, the expression of FUNDC1 and LC3A/B was significantly reduced (*P* < 0.0001), while P62 expression was restored (*P* = 0.0003) (Figs. 5J–K).

These findings suggest that GLU supplementation may alleviate apoptosis and excessive autophagy induced by the co-challenge of PRRSV and DON, both in vivo and in vitro.

### GLU alleviates oxidative stress caused by PRRSV and DON co-challenge

**Figure 6:**
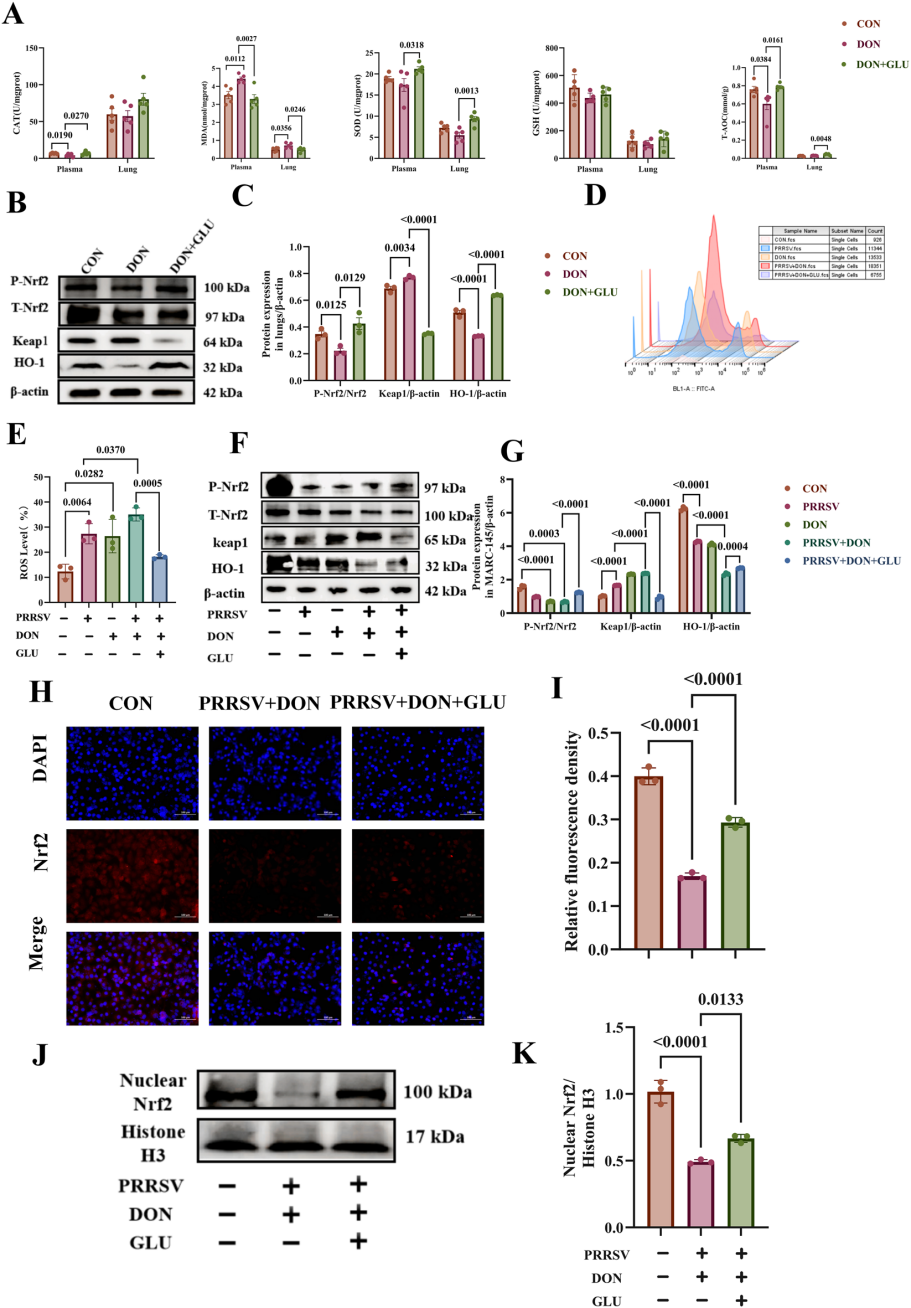
**Glucuronolactone alleviates oxidative stress caused by co-challenge with PRRSV and DON in piglets**. **A** The antioxidant capacity in serum and lung. **B**, **C** Protein expression of Nrf2-related genes by Western Blot in lungs. **D**, **E** Intracellular ROS levels in MARC-145 cells. **F**, **G** Protein expression of Nrf2-related genes by Western Blot in MARC 145 cells. **H**, **I** IFA analysis of Nrf2 (red) using a rabbit Nrf2 protein polyclonal antibody, Scale bar = 100 μm. **J**, **K** Protein levels of cytosolic and Nuclear Nrf2 in the MARC-145 cells. The data are expressed as mean ± SEM (*n* = 3 or 6). CON, control; DON, deoxynivalenol; DON + GLU, combination of deoxynivalenol and glucuronolactone. CAT, catalase; MDA, Malondialdehyde; SOD, Superoxide dismutase; GSH-PX, Glutathione peroxidase; T-AOC, Total antioxidant capacity; Nuclear factor erythroid 2-related factor 2, Nrf2.

We investigated the effect of GLU on oxidative stress induced by co-challenge with PRRSV and DON, which can lead to excessive autophagy. As shown in Fig. 6A, compared to the control group fed the basal diet, the DON group exhibited significantly higher plasma levels of MDA (*P* = 0.0112) and significantly lower levels of CAT (*P* = 0.0190) and T-AOC (*P* = 0.0384). Treatment with additional GLU significantly increased plasma levels of CAT (P = 0.0270), SOD (P = 0.0318), and T-AOC (P = 0.0161), while reducing MDA levels compared to the DON group (*P* = 0.0027).

Similarly, piglets in the DON group exhibited significantly higher levels of MDA in the lungs compared to those in the CON group (*P* = 0.0356). GLU treatment effectively mitigated the elevation of pulmonary MDA levels induced by DON (*P* = 0.0246) and significantly enhanced the levels of SOD (*P* = 0.0013) and T-AOC (*P* = 0.0048) in the lungs compared to the DON group. Additionally, the mRNA expression level of *NQO1* was significantly reduced in the lungs of piglets from the DON group compared to the CON group (*P* = 0.0243) (Additional file 5A).

In contrast, in the DON + GLU group, the mRNA expression levels of *SOD1* (*P* = 0.0040), *GCLM* (*P* = 0.0188), *HO-1* (*P* = 0.0093), and *HMOX* (*P* = 0.0045) were significantly upregulated compared to the DON group (Additional file 5A). The Nrf2 signalling pathway is crucial for modulating oxidative stress [22].

In comparison to the CON group, the DON group showed a significant reduction in the p-Nrf2/Nrf2 (*P* = 0.0125) ratio and HO-1 (*P* < 0.0001) protein levels. Additionally, there was a significant increase in Keap1 (*P* = 0.0034) expression (Figs. 6B and C). Interestingly, in the DON + GLU group, both the p-Nrf2/Nrf2 (*P* = 0.0129) ratio and HO-1 (*P* < 0.0001) protein levels were significantly elevated, while the expression of Keap1 was markedly reduced (*P* < 0.0001).

The excessive accumulation of ROS is a characteristic feature of oxidative stress. We measured intracellular ROS levels using the DCFH2-DA fluorescent probe in combination with flow cytometry. The results indicated that the ROS level was significantly higher in the PRRSV + DON group compared to the PRRSV group (*P* = 0.0370) (Figs. 6D and E). This finding confirms that exposure to DON exacerbates oxidative stress in cells infected with PRRSV. Notably, in the PRRSV + DON + GLU group, the ROS level was significantly lower compared to the PRRSV + DON group (*P* = 0.0005) (Figs. 6D and E). This suggests that GLU effectively mitigates oxidative stress induced by PRRSV and DON at the cellular level.

As shown in Additional file 5B, the PRRSV and DON groups significantly downregulated the mRNA expression level of *NQO-1* (*P* < 0.0001) compared with the CON group. Furthermore, compared to the PRRSV group, the PRRSV + DON group significantly downregulated the mRNA expression levels of *NQO-1* (*P* = 0.0290) and GCLM (*P* = 0.0270). When compared to the PRRSV + DON group, the PRRSV + DON + GLU group significantly increased the mRNA expression levels of intracellular antioxidant-related genes *NQO-1* (*P* = 0.0059), HO-1 (*P* < 0.0001), GCLC (*P* < 0.0001), and *GCLM* (*P* = 0.0022).

To investigate the molecular mechanism underlying the antioxidant effect of GLU, we performed Western blot analysis to evaluate the Nrf2 pathway (Figs. 6F and G). Compared to the CON group, the PRRSV group showed a significant increase in Keap1 protein expression levels and a marked reduction in HO-1 protein expression levels. In contrast, the DON group demonstrated a significant decrease in the P-Nrf2 to Nrf2 ratio (*P* < 0.0001). Furthermore, compared to the PRRSV group, the PRRSV + DON exhibited additional decreases in the p-Nrf2/Nrf2 ratio (*P* = 0.0003) and HO-1 (*P* < 0.0001) protein levels, along with a corresponding increase in Keap1 protein expression (*P* < 0.0001).

In the PRRSV + DON + GLU group, the p-Nrf2/Nrf2 (*P* < 0.0001) ratio and HO-1 (*P* = 0.0004) protein levels were significantly upregulated, while Keap1 expression was notably reduced compared to the PRRSV + DON group (*P* < 0.0001). To further explore the mechanism of GLU-mediated Nrf2 activation, we performed immunofluorescence staining on MARC-145 cells (Figs. 6H and I). The PRRSV + DON group showed significantly weaker fluorescence intensity for Nrf2 localisation in the nucleus compared to the CON group (*P* < 0.0001). As anticipated, in the PRRSV + DON + GLU group, the fluorescence intensity of nuclear Nrf2 was significantly enhanced compared to the PRRSV + DON group (*P* < 0.0001), suggesting that GLU promotes Nrf2 nuclear translocation.

Additionally, Western blot analysis was conducted to confirm the promotion of Nrf2 nuclear translocation by GLU (Figs. 6J and K). Compared to the CON group, the PRRSV + DON group exhibited significantly reduced levels of Nrf2 protein in the nucleus (*P* < 0.0001). In contrast, treatment with GLU resulted in an upregulation of nuclear Nrf2 levels following co-challenge with PRRSV and DON (*P* = 0.0133).

Overall, these findings indicate that GLU supplementation can reduce oxidative stress and activate the Nrf2 pathway in response to PRRSV and DON challenges.

### The specific Nrf2 inhibitor (ML385) negates the therapeutic effects of GLU on PRRSV and DON-induced oxidative stress

The above experimental results demonstrated that GLU treatment effectively activated the Nrf2 signalling pathway under PRRSV and DON co-challenge. To further investigate the role of the Nrf2 signalling pathway in the protective effect of GLU against the injury and inflammation caused by the co-challenge of PRRSV and DON, we employed ML385, a specific Nrf2 inhibitor.

**Figure 7:**
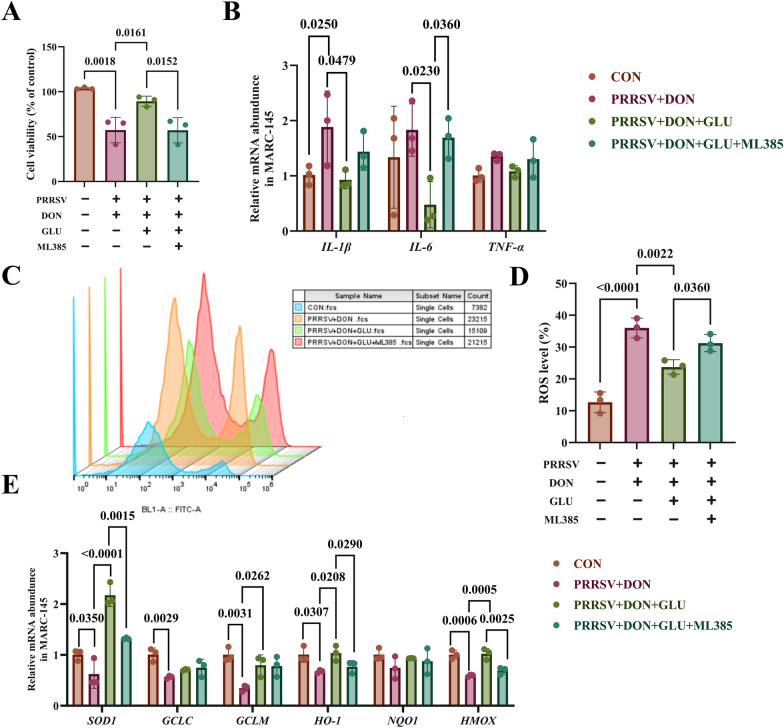
**Specific Nrf2 inhibitor (ML385) inhibited the therapeutic effects of GLU on oxidative stress induced by PRRSV and DON co-challenge**. **A** Cell viability in MARC 145 cells. **B** Cytokine mRNA expression examined by qPCR. **C**, **D** Intracellular ROS levels in MARC-145 cells. **E** mRNA expression of antioxidant genes in the Marc-145 cells. The data are expressed as mean ± SEM (*n* = 3). CON, control; DON, deoxynivalenol; DON + GLU, combination of deoxynivalenol and glucuronolactone. *IL-1β*, Interleukin-1 beta; *IL-6*, Interleukin-6; *IL-8*, Interleukin-8; *TNF-α*, Tumor Necrosis Factor-alpha, *SOD1*, superoxide dismutase; *GCLC*, glutamate-cysteine-ligase cata-lytic subunit; *GCLM*, glutamate-cysteine-ligase modulatory subunit; *HO-1*, hemeoxygenase-1; *NQO-1*, NAD(P)H dehydrogenase, quinone.

As illustrated in Figs. 7A and B, compared to the PRRSV + DON + GLU group, the group treated with ML385 showed significantly reduced cell viability and increased expression of IL-6 (*P* = 0.0360). Furthermore, treatment with ML385 resulted in elevated cellular ROS levels (*P* = 0.0360) (Figs. 7C and D), and decreased mRNA expression of *SOD-1* (*P* = 0.0015), *HO-1* (*P* = 0.0290), and *HMOX* (*P* = 0.0025) (Fig. 7E).

These results suggest that the Nrf2 inhibitor ML385 eliminates the therapeutic effect of GLU on PRRSV and DON co-infection, confirming that GLU mitigates disorders induced by PRRSV and DON co-challenge through the activation of the Nrf2 pathway.

## Discussion

Deoxynivalenol (DON) is a mycotoxin that frequently occurs in feed ingredients and can reach relatively high concentrations, posing a significant risk to livestock health, particularly in swine. Infection with PRRSV can lead to symptoms such as fever and lethargy in pigs. When pigs are co-infected with bacterial pathogens, the situation can worsen, leading to severe pneumonia and a deterioration in overall health [23, 24].

This study emphasises the negative impacts of PRRSV and DON exposure, which significantly worsen both clinical symptoms and growth performance in piglets. DON exposure amplifies the severity of the PRRSV infection, indicating that mycotoxins like DON can intensify the pathological and clinical effects of viral infections.

After being absorbed orally, GLU is metabolised into glucuronic acid, which plays a crucial role in detoxifying harmful substances in the body. It does this by conjugating with hepatically derived toxins and promoting their excretion [25]. While GLU is primarily recognised for its hepatoprotective effects, our study first demonstrates that it also alleviates oxidative stress in the lungs. This reduction in oxidative damage and apoptosis is significant in the context of PRRSV and DON co-challenge.

In previous studies, it has been demonstrated that DON can impair resistance to viral infections in both mice and broilers [26–28]. In our research, we found that DON present in pig feed increased the PRRSV viral load in the blood and lung tissue of pigs. This finding aligns with previous reports indicating that DON raises viral loads in the lungs of mice [26].

However, it is important to note that DON does not directly enhance the replication of PRRSV. Instead, it contributes to heightened inflammatory responses, which are known to worsen clinical outcomes during viral infections [29, 30]. Further research shows that DON can lead to the overexpression of pro-inflammatory cytokines in the lungs of mice [31].

In our study, we observed that DON also stimulated the overexpression of these pro-inflammatory cytokines, resulting in worsened lung pathology. Interestingly, piglets in the DON + GLU group, which received GLU supplementation, exhibited significantly alleviated clinical symptoms, improved growth performance, reduced secretion of inflammatory cytokines, and decreased lung tissue damage. These findings suggest that GLU has a protective effect against PRRSV and DON co-challenge.

Cell apoptosis is typically triggered through three main pathways: the mitochondrial-mediated pathway, the endoplasmic reticulum-mediated pathway, and the death receptor pathway. Among these, the mitochondrial-mediated pathway is the most common trigger [32].

When cells are exposed to external stimuli, the pro-apoptotic protein Bax dissociates from the anti-apoptotic protein Bcl-2 complex and translocates to the outer mitochondrial membrane, where it forms permeability pores. This leads to increased mitochondrial membrane permeability, which activates the apoptotic caspase-3 protein, leading to apoptosis [33, 34].

Research has found that treatment with DON can promote apoptosis by activating the mitochondrial apoptosis pathway. Specifically, DON treatment increases Bax expression while inhibiting Bcl-2 expression, which ultimately results in the activation of caspase-3 and apoptosis [35].

Consistent with these findings, our results demonstrated that co-challenge with PRRSV and DON led to an increase in the expression of mitochondrial apoptosis-related genes and caspase-3 in piglets. Additionally, it has been reported that treatment with GLU resulted in a decrease in caspase-3 mRNA expression and an increase in Bcl-2 mRNA expression when chicken hepatocytes were challenged with OTA [36].

In our study, GLU also reduced cell apoptosis induced by the co-challenge of PRRSV and DON in porcine lung cells.

Apoptosis can be triggered by excessive autophagy [21]. In this study, transmission electron microscopy was used to investigate the effects of co-challenge with PRRSV and DON, revealing damage to the mitochondrial membrane structure. This damage led to excessive autophagy in the type II alveolar epithelial cells of piglets. As a result, excessive autophagy can trigger apoptosis through various mechanisms. For example, it can cause the degradation of essential cellular components, disrupting cellular homeostasis and ultimately leading to apoptosis [37]. The mechanisms underlying autophagy are primarily associated with LC3 and p62 [38]. The co-infection with PRRSV and DON resulted in excessive cellular autophagy, while GLU treatment alleviated this condition by decreasing LC3 expression and increasing p62 expression. Both in vivo and in vitro results confirmed that GLU treatment effectively inhibited the excessive autophagy induced by PRRSV and DON, which may contribute to the reduction of cell apoptosis.

Excessive autophagy, which promotes apoptosis, can be induced by robust oxidative stress [39]. Wu et al. demonstrated that a diet contaminated with 4 mg/kg of DON triggered oxidative stress in piglets. Following the DON challenge, the levels of lipid peroxidation products, such as MDA, were significantly elevated, while the activity of antioxidant-related enzymes was notably reduced [40].

In our study, the experimental results align with these previous findings. Specifically, the co-challenge of PRRSV and DON resulted in a more robust oxidative stress response in piglets, as evidenced by elevated MDA levels in both plasma and lung tissue. Moreover, the activities of antioxidant markers, such as SOD and T-AOC, further decreased after co-challenging with PRRSV and DON. Thus, alleviating oxidative stress presents a potential therapeutic strategy for improving overall body health.

In an in vitro model using human colon cancer HT-29 cells, the addition of natural antioxidants, such as epigallocatechin-3-gallate and lutein, effectively reduced DON-induced cellular oxidative stress. This addition increased cellular GSH levels and decreased cellular ROS levels, leading to improvements in cell viability [41, 42].

Our study found that supplementing piglets’ diets with GLU effectively inhibited oxidative stress and enhanced the activities of antioxidant-related enzymes in the plasma and lung tissue of piglets co-infected with PRRSV and DON. Additionally, GLU significantly suppressed the increase in ROS levels caused by PRRSV and DON in MARC-145 cells. Therefore, the antioxidant GLU could help alleviate PRRSV and DON in piglets by enhancing antioxidant capacity and reducing oxidative stress levels.

Nrf2 plays a critical role in cellular defence, particularly in regulating genes involved in the oxidative stress response, such as HMOX1, NQO1, GCLC, and GCLM [43]. This study investigated the expression of antioxidant-related genes and proteins that are downstream of the Nrf2 signalling pathway through both in vivo and in vitro experiments. The objective was to assess the impact of co-challenging with PRRSV and DON, as well as GLU treatment, on the activity of the Nrf2 signalling pathway.

The experimental results indicated that co-challenge with PRRSV and DON could further reduce the activity of the Nrf2 pathway and the expression of antioxidant-related genes in porcine lung tissue. Chen et al. demonstrated that glucuronolactone has strong antioxidant properties, enhancing the activity of SOD and GSH activity, and alleviating liver injury in rats [44].

Consistently, our results showed that adding GLU to the diet effectively mitigated the suppression of the Nrf2 signalling pathway caused by PRRSV and DON, resulting in increased expression levels of downstream antioxidant-related genes and proteins. In this study, we demonstrated that dietary GLU activation of the Nrf2 signalling pathway enhances antioxidant capacity, thereby alleviating oxidative damage induced by PRRSV and DON in porcine lung tissue.

In our study, we utilised ML385, an inhibitor of the Nrf2 protein, to investigate whether GLU resistance to oxidative stress is dependent on the Nrf2-mediated signalling pathway. Our findings revealed that the use of ML385 nearly completely negated the protective effects of GLU. Additionally, there was no significant difference in the ROS content and downstream antioxidant gene expression in MARC-145 cells between the PRRSV + DON group and the PRRSV + DON + GLU + ML385 group. Therefore, it can be concluded that GLU’s protective effect against oxidative damage induced by PRRSV and DON relies on the Nrf2-mediated signalling pathway.

Several limitations should be considered when interpreting these findings. First, the use of a single time point in our design prevents us from analysing the temporal dynamics between DON exposure and the progression of PRRSV. Second, while oxidative stress seems to play a central role in the interactions between DON and PRRSV, there may be unexplored mechanisms that contribute synergistically. For example, investigating the direct molecular interactions between Nrf2 and regulators of apoptosis and autophagy could provide additional insights. Third, since we used the GD-ZJ strain in our research, we should exercise caution when generalising the results across the genetically diverse populations of PRRSV. Addressing these limitations through multi-omics approaches, longitudinal sampling, and comparative studies with different strains will be crucial for future research.

Our study demonstrates that GLU supplementation effectively enhances growth performance and reduces pathological damage caused by the co-challenge of PRRSV and DON in piglets. GLU supplementation promotes the phosphorylation and nuclear import of Nrf2, helping to alleviate oxidative stress induced by this co-challenge. This process contributes to the inhibition of excessive autophagy and apoptosis. The use of the Nrf2 inhibitor ML385 confirms the essential role of Nrf2 in mediating the protective effects of GLU, suggesting that Nrf2 may be a promising therapeutic target for mitigating the negative impacts of viral and mycotoxin co-challenge in the pig industry.

## Supplementary Information

**Additional file 1. Dietary ingredient composition in animal experiment.**

**Additional file 2. The levels of main mycotoxins in feed.**

**Additional file 3. Primers used in this study.**

**Additional file 4. Biochemical indicators of liver and kidney function in different groups.** ^1.^Values are presented as means and pooled SEM, *n* = 6/treatment; CON, control; DON, deoxynivalenol; DON+GLU, combination of deoxynivalenol and glucuronolactone; URE, urea; CRE, creatinine; GLU, glucose; AST, aspartate aminotransferase; ALT, alanine aminotransferase; TP, total protein; ALB, albumin.

**Additional file 5. Protective effect of GLU on inflammation induced by PRRSV and DON co-challenge in vivo and in vitro.** **A** Effect of DON on cell viability following PRRSV infection. **B** Effect of glucuronolactone on cell viability. **C** 50% cytotoxic concentration analysis. Values are presented as means and pooled SEM, *n* = 3/treatment; DON, deoxynivalenol; GLU, glucuronolactone.

**Additional file 6. GLU alleviates oxidative stress caused by PRRSV and DON co-challenge.** **A** mRNA expression of antioxidant genes in the lungs of piglets co-challenged with PRRSV and DON. **B** Effect of GLU on mRNA expression of antioxidant genes in MARC-145 cells co-infected with PRRSV and DON. Data are presented as means and pooled SEM, *n* = 3 or 6; CON, control; DON, deoxynivalenol; DON+GLU, combination of deoxynivalenol and glucuronolactone; *SOD1*, superoxide dismutase; *GCLC*, glutamate-cysteine-ligase catalytic subunit; *GCLM*, glutamate-cysteine-ligase modulatory subunit; *HO-1*, hemeoxygenase-1; *NQO-1*, NADH dehydrogenase, quinone.

## Data Availability

The data supporting the findings of this study are available from the corresponding author upon reasonable request.
