# Supplementary material for: Glucuronolactone improves lung injury caused by PRRSV and DON co-challenge by enhancing the Nrf2-mediated antioxidant capacity in weaning piglets
Source: Vet Res. 2025 Aug 5;56:161. doi: 10.1186/s13567-025-01596-8 (PMC12326727; doi:10.1186/s13567-025-01596-8)
Supplement: Supplementary file 1 — Additional file 1. Dietary ingredient composition in animal experiment. [file 13567_2025_1596_MOESM1_ESM.docx]

**Additional file 1 Dietary ingredient composition in animal experiment**

| Ingredients | Amount (%) | Nutrient levels | Amount (%) |
| --- | --- | --- | --- |
| Corn | 53.87 | Net energy (kcal/kg) | 2550 |
| Soybean meal | 25.10 | Crude protein | 17.56 |
| Low protein whey powder | 6.00 | SID crude protein | 1.35 |
| Whey protein concentrate | 3.00 | SID lysine | 0.74 |
| Fish meal | 3.00 | SID methionine + cysteinesides threonine | 0.80 |
| Soybean oil | 2.50 | SID tryptophan | 0.24 |
| Sucrose | 2.00 | SID isoleucine | 0.80 |
| L-Lys•HCl | 0.35 | SID valine | 0.86 |
| DL-Met | 0.13 | SID leucine | 1.60 |
| L-Thr | 0.10 | SID Lysine/net energy (g/MJ) | 5.27 |
| CaHPO4 | 0.80 | Total calcium | 0.75 |
| Calcium citrate | 0.50 | Total phosphorus | 0.61 |
| Stone powder | 0.50 | STTD P | 0.39 |
| NaCl | 0.35 |  |  |
| ZnO | 0.18 |  |  |
| TiO2 | 0.40 |  |  |
| Choline chloride | 0.20 |  |  |
| Phytase | 0.02 |  |  |
| Premix | 1.00 |  |  |
| Total | 100 |  |  |
