# Supplementary material for: Glucuronolactone improves lung injury caused by PRRSV and DON co-challenge by enhancing the Nrf2-mediated antioxidant capacity in weaning piglets
Source: Vet Res. 2025 Aug 5;56:161. doi: 10.1186/s13567-025-01596-8 (PMC12326727; doi:10.1186/s13567-025-01596-8)
Supplement: Supplementary file 2 — Additional file 2. The levels of main mycotoxins in feed. [file 13567_2025_1596_MOESM2_ESM.docx]

Additional file 2 The levels of main mycotoxins in feed

| Item | Basal feed | Contaminated feed | Limit of detection | Dose limited in China |
| --- | --- | --- | --- | --- |
| DON (μg/kg) | 200 | 2000 | 100 | 1000 |
| AFB1 (μg/kg) | undetected | undetected | 2 | 10 |
| ZEN (μg/kg) | undetected | undetected | 10 | 150 |
| OTA (μg/kg) | undetected | undetected | 10 | 100 |
| T2 (μg/kg) | undetected | undetected | 100 | 500 |
