# Supplementary material for: Glucuronolactone improves lung injury caused by PRRSV and DON co-challenge by enhancing the Nrf2-mediated antioxidant capacity in weaning piglets
Source: Vet Res. 2025 Aug 5;56:161. doi: 10.1186/s13567-025-01596-8 (PMC12326727; doi:10.1186/s13567-025-01596-8)
Supplement: Supplementary file 3 — Additional file 3. Primers used in this study. [file 13567_2025_1596_MOESM3_ESM.docx]

Additional file 3 Primers used in this study (For sus scrofa).

| Genes | Forward | Reverse |
| --- | --- | --- |
| *β-actin* | TGTGGATCAGCAAGCAGGAG | GAATGCAACTAACAGTCCGCC |
| *IL-1β* | CTCCAGCCAGTCTTCATTGTTC | TGCCTGATGCTCTTGTTCCA |
| *IL-6* | TACATCCTCGGCAAAATC | TCTCATCAAGCAGGTCTCC |
| *IL-10* | CTGCATCCACTTCCCAACCA | CGGCATTACGTCTTCCAGGT |
| *TNF-α* | CCAATGGCAGAGTGGGTATG | TGAAGAGGACCTGGGAGTAG |
| *IFN-α* | TCACAGAGTCACCCACCTCA | CCCAGAGAGCAGATGGCATT |
| *IFN-β* | GCCCACCAACTCAGCATTTG | AAGCCAGTAGGCACTTTGGG |
| *Bax* | GACGCTGGACTTCCTTCGAG | GTGGCCCGAGAGAGGTTTATT |
| *Bcl-2* | GCTACTTACTGCCAAAGGGA | TTCAGGCGGAGCTGTAAGAG |
| *Caspase-3* | GCAGAATTGACCCTCACGAT | GCAGAATTGACCCTCACGAT |
| *Caspase-8* | TCTGCGGACTGGATGTGATT | TCTGAGGTTGCTGGTCACAC |
| *Caspase-9* | AATGCCGATTTGGCTTACGT | CATTTGCTTGGCAGTCAGGTT |
| *SOD1* | GAGACCTGGGCAATGTGACT | CTGCCCAAGTCATCTGGTTT |
| *GCLC* | CAAACCATCCTACCCTTTGG | ATTGTGCAGAGAGCCTGGTT |
| *GCLM* | GATGCCGCCCGATTTAACTG | ACAATGACCGAGTACCGCAG |
| *HO-1* | CGCTCCCGAATGAACACTCT | GCGAGGGTCTCTGGTCCTTA |
| *NQO-1* | ATCACAGGTAAACTGAAGGACCC | TGGCAGCGTATGTGTAAGCA |
| *HMOX* | CGCTCCCGAATGAACACTCT | GCGAGGGTCTCTGGTCCTTA |
| *ORF 7* | CCAGCCGGTCAATCAGCT | GGCTTCTCCGGGCTTTTCT |

^1^*IL-1β*, Interleukin-1 beta; *IL-6*, Interleukin-6; *TNF-α*, Tumor Necrosis Factor-alpha; *IFN-α*, Interferon-alpha; *IFN-β*, Interferon-beta; *IL-10*, Interleukin-10; *Bax*, Bcl-2-associated X protein; *Bcl-2*, B-cell lymphoma 2. *SOD1*, superoxide dismutase; *GCLC*, glutamate-cysteine-ligase cata-lytic subunit; *GCLM*, glutamate-cysteine-ligase modulatory subunit; *HO-1*, hemeoxygenase-1; *NQO-1*, NAD(P)H dehydrogenase, quinone.

Primers used in this study (For green monkey).

| Genes | Forward | Reverse |
| --- | --- | --- |
| *β-actin* | ATCTGGCACCACACCTTCTACAATGAGCTGCG | CGTCATACTCCTGCTTGCTGATCCACATCTG |
| *IL-1β* | GAGTCTGCCCAATTCCCCAA | ATATCCTGGCCACCTCTGGT |
| *IL-6* | ACTCCCTCTCCACAAGCGCCT | TGGCATCTTCTTCCAGGCGTCCC |
| *TNF-α* | GCCCACGTTGTAGCCAATGTCAAA | GTTGTCTTTCAGCTTCACGCCGTT |
| *PRRSV-N* | GAG ATG ATC CAG TTT TAC CTGG | CGGATCAGACGCACAGTATG |
| *Bax* | AGATTTGCGATTGGACGGGT | TGTCCAGCCCATGATGGTTC |
| *Bcl-2* | GGCCGGACTAACTCACCATT | TGATGCCGAAGTCACCGAAA |
| *Caspase-3* | TGAGGAGGCTGTGCGTTAAT | TCGGCGTACTGTTTCAGCAT |
| *Caspase-8* | TCAGGCTTGTCAGGGGGATA | TCCCAAAGCCCTAGGCATAAGA |
| *Caspase-9* | ACTCGAGGGAGTCAAGCTCT | TCTTTCTGCTCAACATCACCGA |
| *SOD1* | GAGACCTGGGCAATGTGACT | CTGCCCAAGTCATCTGGTTT |
| *GCLC* | ACATGCGAAAACGGCGGAA | CGAGGGTGCTTGTTTATT GC |
| *GCLM* | TCAGTGGGCACAGGTAAAA | TTGTTTAGCAAATGCAGTCA |
| *HO-1* | CTTCAAGCTGGTGATGGC | TGGAGCCGCTTCACATAG |
| *NQO-1* | CATGTACTCTCTGCAAGGGA | TCCCAAATATTCTCCAGGCG |
| *HMOX* | CTTCAAGCTGGTGATGGC | TGGAGCCGCTTCACATAG |
| *ORF 7* | CCAGCCGGTCAATCAGCT | GGCTTCTCCGGGCTTTTCT |

^1^*IL-1β*, Interleukin-1 beta; *IL-6*, Interleukin-6; *TNF-α*, Tumor Necrosis Factor-alpha; *IFN-α*, Interferon-alpha; *IFN-β*, Interferon-beta; *IL-10*, Interleukin-10; *Bax*, Bcl-2-associated X protein; *Bcl-2*, B-cell lymphoma 2. *SOD1*, superoxide dismutase; *GCLC*, glutamate-cysteine-ligase cata-lytic subunit; *GCLM*, glutamate-cysteine-ligase modulatory subunit; *HO-1*, hemeoxygenase-1; *NQO-1*, NAD(P)H dehydrogenase, quinone.
