# Supplementary figures and images for: Glucuronolactone improves lung injury caused by PRRSV and DON co-challenge by enhancing the Nrf2-mediated antioxidant capacity in weaning piglets
Source: Vet Res. 2025 Aug 5;56:161. doi: 10.1186/s13567-025-01596-8 (PMC12326727; doi:10.1186/s13567-025-01596-8)

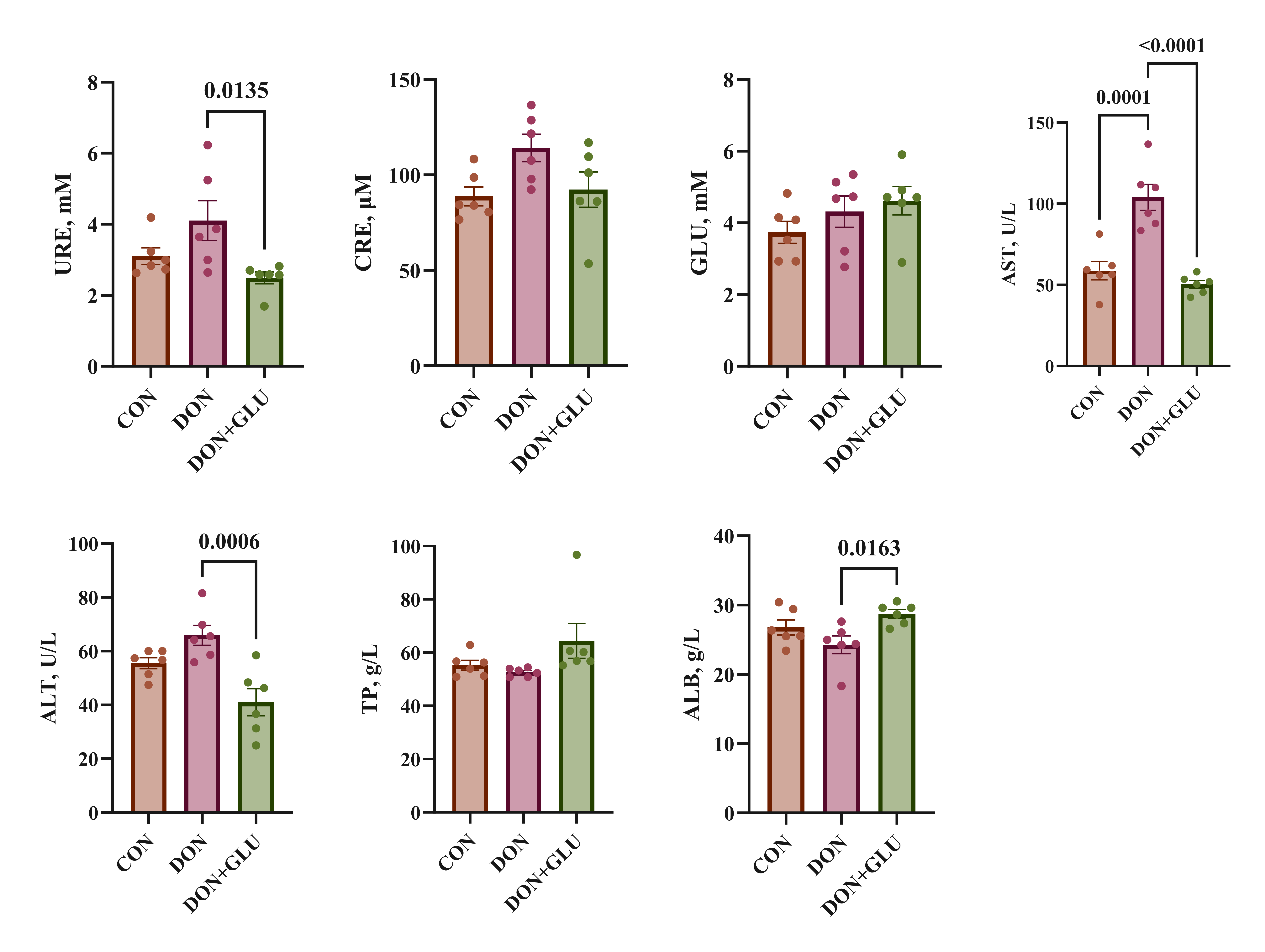

Supplement: Supplementary file 4 — Additional file 4. Biochemical indicators of liver and kidney function in different groups. 1.Values are presented as means and pooled SEM, n = 6/treatment; CON, control; DON, deoxynivalenol; DON+GLU, combination of deoxynivalenol and glucuronolactone; URE, urea; CRE, creatinine; GLU, glucose; AST, aspartate aminotransferase; ALT, alanine aminotransferase; TP, total protein; ALB, albumin. [file 13567_2025_1596_MOESM4_ESM.tif]

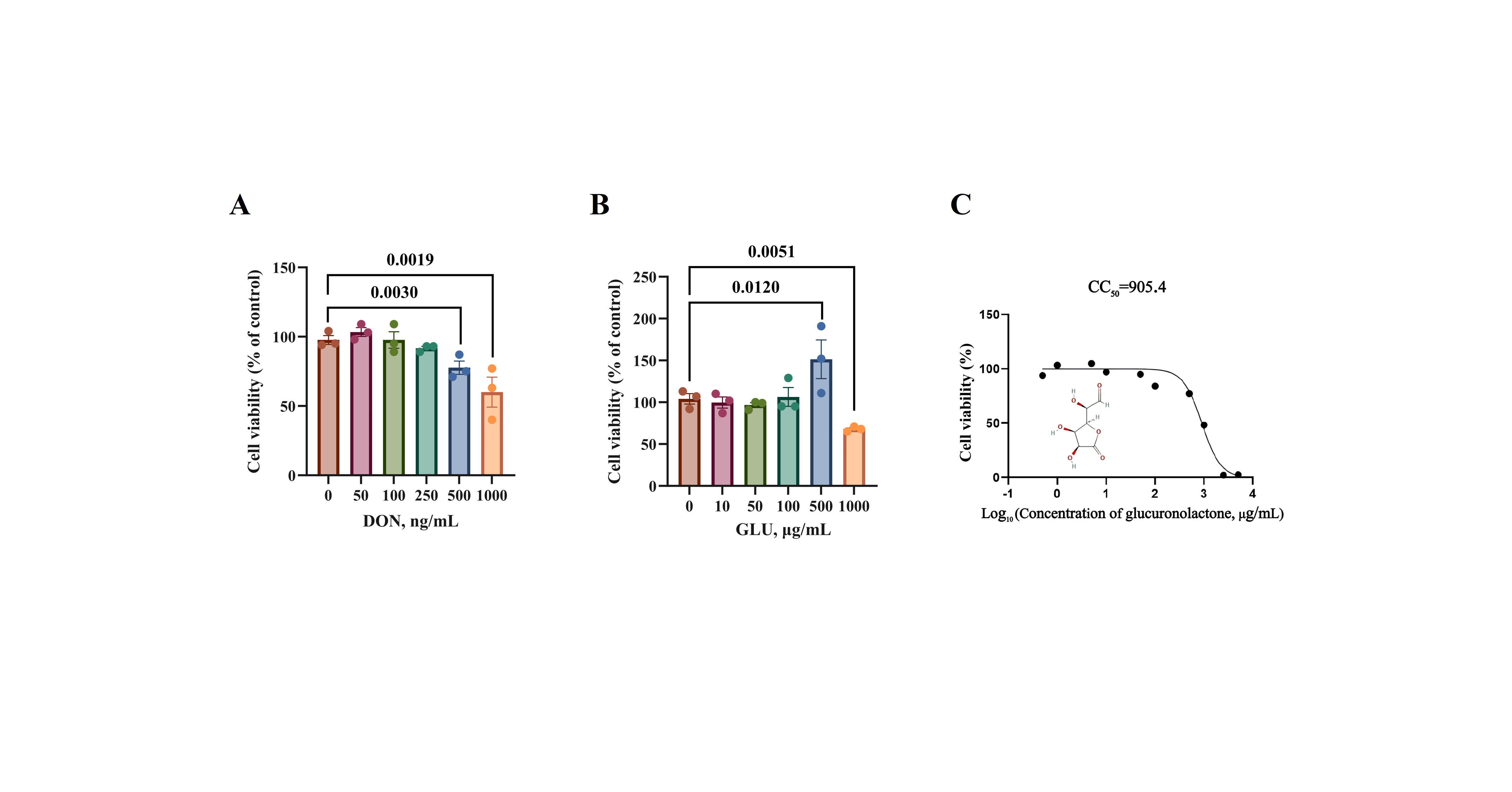

Supplement: Supplementary file 5 — Additional file 5. Protective effect of GLU on inflammation induced by PRRSV and DON co-challenge in vivo and in vitro. A Effect of DON on cell viability following PRRSV infection. B Effect of glucuronolactone on cell viability. C 50% cytotoxic concentration analysis. Values are presented as means and pooled SEM, n = 3/treatment; DON, deoxynivalenol; GLU, glucuronolactone. [file 13567_2025_1596_MOESM5_ESM.tif]
